# Managed retreat through voluntary buyouts of flood-prone properties

**DOI:** 10.1126/sciadv.aax8995

**Published:** 2019-10-09

**Authors:** Katharine J. Mach, Caroline M. Kraan, Miyuki Hino, A. R. Siders, Erica M. Johnston, Christopher B. Field

**Affiliations:** 1 Rosenstiel School of Marine and Atmospheric Science, University of Miami, Miami, FL 33149, USA.; 2 Leonard and Jayne Abess Center for Ecosystem Science and Policy, University of Miami, Coral Gables, FL 33124, USA.; 3 Department of Earth System Science, Stanford University, Stanford, CA 94305, USA.; 4 Stanford Woods Institute for the Environment, Stanford University, Stanford, CA 94305, USA.; 5 Leonard and Jayne Abess Center for Ecosystem Science and Policy, Environmental Science and Policy Graduate Program, University of Miami, Coral Gables, FL 33124, USA.; 6 Emmett Interdisciplinary Program in Environment and Resources, Stanford University, Stanford, CA 94305, USA.; 7 Center for the Environment, Harvard University, Cambridge, MA 02138, USA.; 8 Disaster Research Center, Biden School of Public Policy and Administration, Geography and Spatial Sciences, University of Delaware, Newark, DE 19716, USA.

## Abstract

Retreat from some areas will become unavoidable under intensifying climate change. Existing deployments of managed retreat are at small scale compared to potential future needs, leaving open questions about where, when, and how retreat under climate change will occur. Here, we analyze more than 40,000 voluntary buyouts of flood-prone properties in the United States, in which homeowners sell properties to the government and the land is restored to open space. In contrast to model-based evaluation of potential future retreat, local governments in counties with higher population and income are more likely to administer buyouts. The bought-out properties themselves, however, are concentrated in areas of greater social vulnerability within these counties, pointing to the importance of assessing the equity of buyout implementation and outcomes. These patterns demonstrate the challenges associated with locally driven implementation of managed retreat and the potential benefits of experimentation with different approaches to retreat.

## Teaser

Experiences with voluntary buyouts of flood-prone properties provide lessons for managed retreat under climate change.

## INTRODUCTION

Development patterns and climate change are together increasing flood risk in many regions around the world (*1*–*4*). Given costs of protection (e.g., coastal armoring) and limits to accommodation (e.g., infrastructure elevation), experts agree that retreat from some areas will become an unavoidable option under intensifying climate change (*5*). However, substantial uncertainty remains regarding which areas will experience retreat and how it will occur. Studies of economically robust coastal adaptation suggest that retreat will take place in lower-income, more rural areas (*6*, *7*), but case studies within the United States have primarily documented retreat in urban areas [e.g., (*8*–*11*)], raising questions about whether predicted patterns of rural retreat will hold true within or across nations. Existing retreat experiences may also offer strategies relevant across international contexts and lessons about revisions needed to meet future demands.

**Fig. 1:**
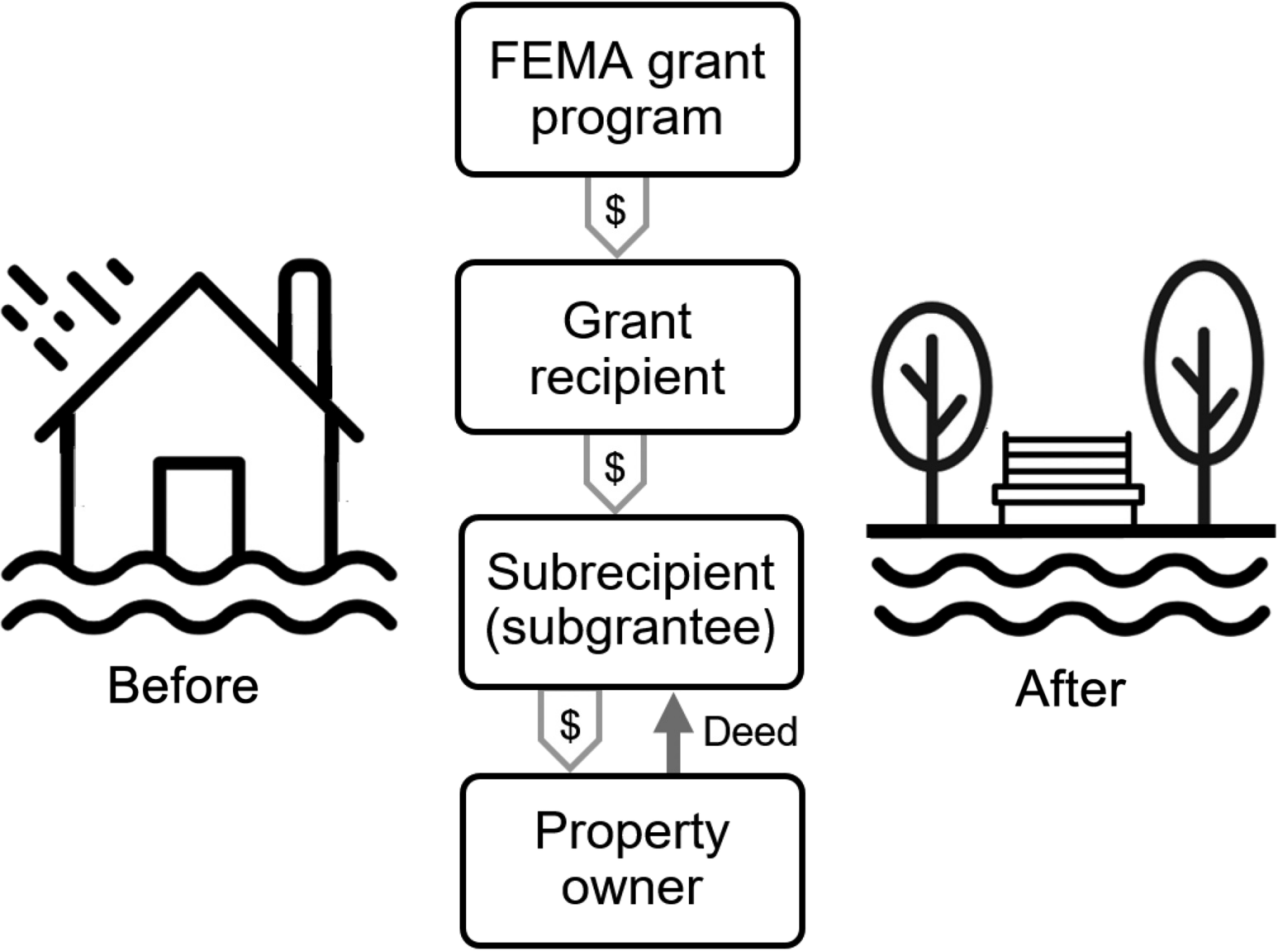
FEMA-funded voluntary property buyouts to manage flood risk. FEMA supports buyouts of flood-prone properties through several grant programs. Under FEMA regulations, the property acquisitions are voluntary. Property owners, generally homeowners, must agree to sell their properties, and eminent domain or condemnation powers cannot be used. Grant applications to FEMA are submitted via states, territories, or federally recognized tribes (“grant recipient” in figure). The buyouts are administered by subgrantees, which are most often local governments (i.e., city or county governments). Subgrantees can also include state agencies, federally recognized tribes, tribal agencies, and private nonprofits. After a buyout, the land is maintained as open space. Icons, modified in figure, are from thenounproject.com.

U.S. programs of managed retreat to reduce natural hazard risk are among the longest-running programs globally (*12*). The predominant means of government-sponsored retreat in the United States has been through voluntary buyouts of flood-prone properties (*13*, *14*). Government acquisition of flood-prone properties has generally been funded by federal agencies, especially the U.S. Federal Emergency Management Agency (FEMA), and administered by state or local governments (Fig. 1). Other agencies, such as the Small Business Administration and the Department of Housing and Urban Development, have also funded floodplain property acquisitions. Furthermore, some buyout programs have been entirely state or locally funded. The focus of this analysis is the FEMA-funded buyouts for which data are publicly and readily available. Under FEMA regulations, project scoping involves consideration of technical feasibility, costs and cost-effectiveness, and environmental, cultural, and community dimensions (*15*). Property owners must agree to sell their properties, and following acquisition, residents relocate, properties are demolished or, in some cases, relocated, and the land must then be maintained as open space in perpetuity to restore floodplain functions. Applicable flood-related hazards include flows or accumulation of water, flows of mud, and, in some cases, erosion, and we adopt this broad definition in analysis of flood-related hazards and flood-prone properties.

Buyouts as a means of accomplishing managed retreat have received a growing amount of scholarly attention (*14*, *16*, *17*). This work, mostly case based, has evaluated factors influencing individual homeowners’ decisions to accept or reject buyout offers (*10*, *18*–*20*), potentially inequitable implementation practices (*9*, *11*), land use after buyouts occur (*14*), relocation outcomes for residents (*21*), and the limited evidence of policy learning through time (*16*).

These studies have questioned whether U.S. federal buyout programs are fair and effective, but the findings have been limited to individual cases and contexts, rather than evaluated at a programmatic level. Empirical analysis of the FEMA buyout funding programs as a whole remains sparse, despite its importance in understanding programmatic efficiency as well as equity. Moreover, while research has explored why individuals accept or reject buyout offers (*18*–*20*), little work has evaluated why local and state governments choose to use buyouts to manage flood risk. Furthermore, recent research suggests that post-disaster aid widens wealth inequality (*22*), and buyouts case studies have raised questions about practices and outcomes in minority and low-income areas (*9*, *11*). Understanding equity issues in post-disaster buyouts is pressing at the programmatic scale, given the potential for systematic inequalities.

Here, we analyze all FEMA-funded voluntary buyouts of flood-prone properties. We build upon hypotheses generated from case-based research to identify broader patterns regarding where and how buyouts occur. Attuned to effectiveness, efficiency, and equity of managed retreat in the United States to date, our assessment determines (i) where voluntary buyouts of flood-prone properties take place, (ii) what flood-related hazards and damage occur in areas with and without buyouts, and (iii) what socioeconomic and demographic characteristics predict which jurisdictions implement buyouts and which neighborhoods are bought out.

This crosscutting approach bolsters empirical foundations for future managed retreat and informs global discussions about efficient and equitable allocation of resources for adaptation. The analyses draw from multiple data sources (see Materials and Methods): OpenFEMA datasets, the SHELDUS version 16.1 database of flood-related property damage, 100-year flood zones from the recently updated river and rainfall-driven flood hazard maps developed by Wing *et al*. (*4*, *23*), and socioeconomic and demographic indicators derived from the U.S. Census American Community Survey (*24*).

## RESULTS

### Spatial and temporal trends in buyouts

**Fig. 2:**
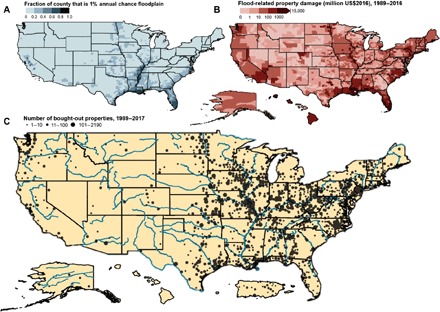
Flood risk and voluntary property buyouts across the United States. (**A**) Exposure to riverine and rainfall-driven flood hazards. The fraction of each county’s area that is 1% annual chance floodplain is shown. (**B**) Flood-related property damage. For each county, cumulative damage over 1989–2016 is depicted (as million US$2016). (**C**) Buyouts of flood-prone properties. The number of FEMA-funded voluntary property buyouts in each county is shown for program years 1989–2017 (as cumulative no. of bought-out properties). Major river systems are illustrated. Insets, not to scale, show available data for Alaska, Hawaii (no bought-out properties), and Puerto Rico.

**Fig. 3:**
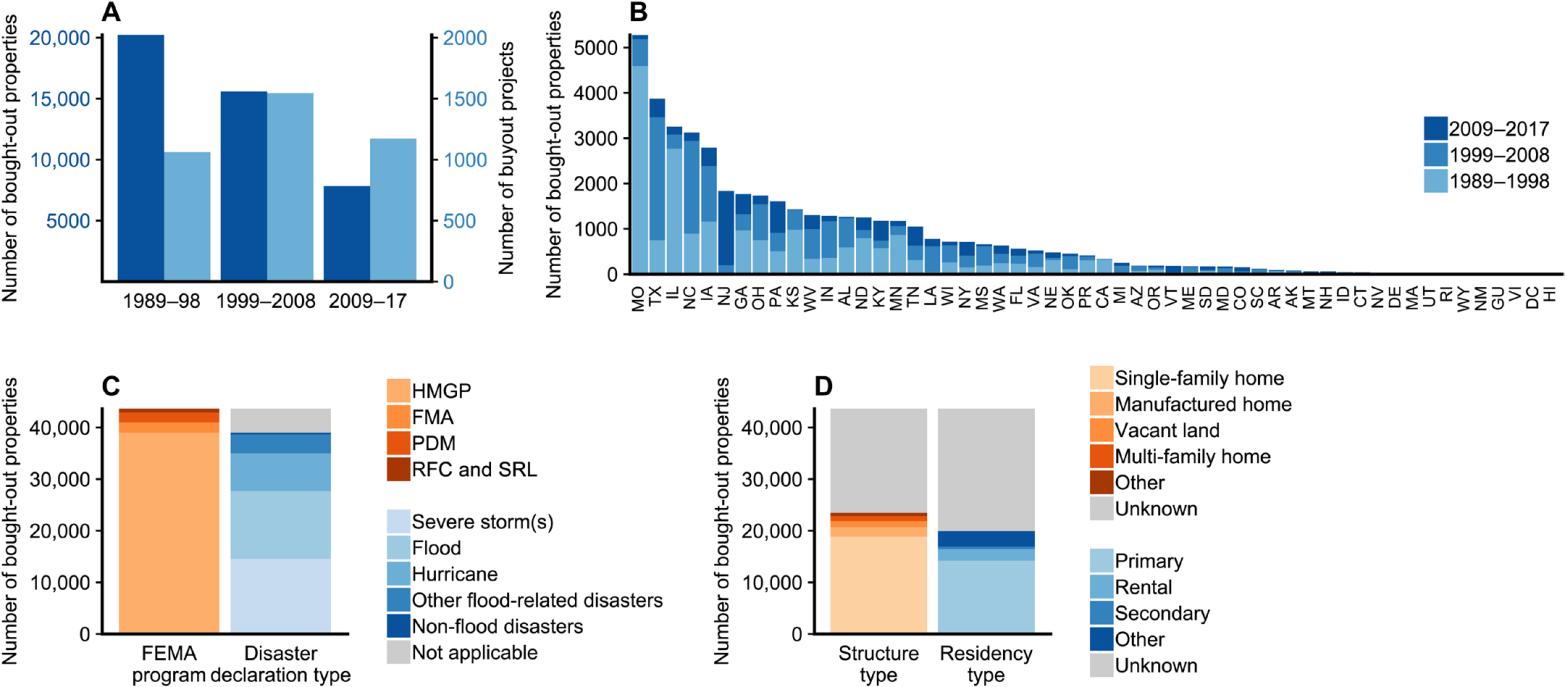
Trends in FEMA-funded buyouts of flood-prone properties over program years 1989–2017. (**A**) Number of bought-out properties and buyout projects, by decade. (**B**) Number of bought-out properties in U.S. states and territories, by decade of buyout-project program years. (**C**) Number of bought-out properties under each relevant FEMA grant program and federal disaster declaration incident type. Relevant FEMA grant programs: Hazard Mitigation Grant Program (HMGP), Flood Mitigation Assistance Grant Program (FMA), the Pre-Disaster Mitigation Grant Program (PDM), the Repetitive Flood Claims Grant Program (RFC), and the Severe Repetitive Loss Grant Program (SRL). (**D**) Structure and residency types of bought-out properties, where specified.

From 1989 to 2017, FEMA funded 43,633 voluntary buyouts of flood-prone properties for which data are publicly available (Figs. 2 and 3). Property buyouts occurred in 49 U.S. states and three U.S. territories (Puerto Rico, Guam, and the U.S. Virgin Islands) (Figs. 2C and 3B). These buyouts have taken place in 1148 counties, where “counties” include county-equivalent entities such as parishes, boroughs, and independent cities. The number of bought-out properties in a single county ranges from 1 to 2190 (median, 11). Nine counties across Texas, Missouri, Alabama, New Jersey, and North Carolina have each bought out more than 500 properties, with greatest deployment in Harris County, Texas (2190 bought-out properties), and St. Charles County, Missouri (1429). One hundred counties have had at least 100 properties bought out, accounting for 24,733 bought-out properties in total. By contrast, 560 counties have had 10 or fewer properties bought out, amounting to 1943 bought-out properties in total.

The locations of property buyouts broadly reflect locations with flood-related hazards, property damage, and disaster declarations (Figs. 2 and 3Band figs. S1 to S3). The six states with the most bought-out properties are all among the top 10 states for cumulative flood-related property damage over the same time frame, in total or per capita (Fig. 3B and fig. S3). However, not all states with high flood-related damages have prioritized buyouts. For example, Florida, Louisiana, and Mississippi have the most cumulative flood-related property damage, but they rank 23, 18, and 21, respectively, for statewide deployment of property buyouts.

Temporal patterns of buyouts vary by state, mirroring temporal shifts in flood damage across the nation. In Midwestern states, most property buyouts took place in the first and second decades of FEMA grant programs supporting buyouts (1989–2008), and most flood-related damage in the Midwest occurred during the same decades (Fig. 3B). By contrast, many New England states have experienced an uptick in buyouts in the most recent decade (2009–2017), coinciding with a rise in flood-related damage in the region.

The number of properties purchased for buyouts has declined in each successive decade (Fig. 3A and fig. S4). By far, 1993 was the year with the greatest number of bought-out properties: more than 8000 in total (fig. S4). However, the number of FEMA grants to support buyouts has remained steady (each unique grant is considered a “buyout project”), and therefore, each project has purchased fewer properties (Fig. 3A and figs. S4 and S5). In 1989–1998, buyout projects acquired a mean of 19 properties (range, 1 to 1410 properties; median, 6 properties). By 2009–2017, the mean size decreased to seven properties (range, 1 to 198 properties; median, 2 properties). Across all three decades 1989–2017, the 3780 buyout projects have had a mean size of 11 properties (range, 1 to 1410 properties; median, 3 properties). Many counties use buyouts only once. Counties with buyouts have had a median of two buyout grant projects each. The greatest number of projects (59) occurred in Harris County, Texas.

### Exposure to flood-related hazards

The Hazard Mitigation Grant Program (HMGP) is the longest-running FEMA program providing grants for voluntary buyouts of flood-prone properties. Subgrantees administering buyout projects are eligible to receive HMGP funds only following a federal disaster declaration. Several types of disasters have historically established eligibility for HMGP funding for property buyouts (Fig. 3C), including severe storms (14,566 bought-out properties), floods (13,170), hurricanes (7283), and other flood-related disasters (3681). In addition, HMGP has funded buyouts of 297 flood-prone properties following non–flood-related disasters, and other FEMA grant programs have funded the remaining 4636 property buyouts.

**Fig. 4:**
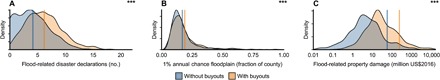
Flood-related exposure in counties in which voluntary property buyouts have versus have not occurred. Flood-related exposure is shown by (**A**) number of flood-related disaster declarations including individual assistance in the county over 1989–2017, (**B**) fraction of county area that is 1% annual chance floodplain, and (**C**) cumulative flood-related property damage over 1989–2016 (as million US$2016). Within each panel, the density plot and mean are orange for counties in which voluntary property buyouts have been administered by any subgrantees over program years 1989–2017, whereas they are blue for counties in which buyouts have not occurred. ***P ≤ 0.001 for Welch’s unequal variances *t* test for differences in means. Data are included for all geographic regions available for each flood-related measure: (A to C) continental United States; (A and C) Alaska and Hawaii; (A) Puerto Rico. The number of counties relevant to each panel is therefore as follows: (A) 3220 counties (2072 in blue, without buyouts and 1148 in orange, with buyouts), (B) 3108 counties (1993 blue and 1115 orange), and (C) 3142 counties (2019 blue and 1123 orange).

Given FEMA’s eligibility requirements, we hypothesize that buyouts have occurred in areas with high levels of flood hazard or damage. This is supported. Counties in which buyouts have occurred have experienced more flood-related disaster declarations and property damage compared to counties in which buyouts have not occurred (Fig. 4 and fig. S6). Counties with buyouts also have more exposure to 1% annual chance floods (Fig. 4B). These patterns hold for coastal and inland counties and for counties with few versus many buyouts evaluated separately. Recent experiences with floods are important: There is more flood-related property damage in the year a buyout project begins, compared to the preceding years (fig. S6).

The types of properties acquired affect the nature of implementation and the risk reduction outcomes of a buyout program. Bought-out properties reflect a range of different structure and residency types (Fig. 3D), but most are single-family structures (18,881 properties) and primary residences (14,248). The next most common structure types include manufactured homes (i.e., mobile homes; 1837 properties), vacant land (1183), and multifamily homes (953). For residency type, rentals (2217 properties) and secondary residences (477) are also represented, although less frequently than primary residences. As a limit for this analysis, structure and residency types are often not recorded. For 20,143 properties (46%) in the FEMA dataset, structure type is not reported, and for 23,676 properties (54%), residency type is not reported.

### Socioeconomics and demographics of implementers and residents

Our analysis of which jurisdictions implement buyouts and, in turn, which neighborhoods are bought out considers three hypotheses emerging from existing literature. First, model-based evaluation of economically robust coastal adaptation predicts that retreat will occur in rural, low-income areas (*6*, *7*). Our findings outlined below only partially support this pattern and depend on the scale of analysis. Second, on the basis of previous buyouts case studies of the difficulties of administering buyouts (*11*, *16*, *25*, *26*), we hypothesize that buyouts will occur in areas with high local government capacity. At the county level, this expectation is supported using income and population as proxies for local government capacity (e.g., presuming wealthier, denser counties have greater funding and staffing for local government). Third, on the basis of the case study descriptions of buyouts in marginalized communities (*8*, *11*, *21*, *26*–*28*), we hypothesize that buyouts will occur in neighborhoods with greater socioeconomic vulnerability. At the subcounty ZIP code level, this expectation is supported.

A total of 94% of the property buyouts (40,898 bought-out properties) were administered by a local government subgrantee: city or county government. In considering the capacity and willingness of the local government to apply for, receive, and administer federal funding, our analysis evaluates county-scale socioeconomic and demographic indicators, as these can be systematically compared across subgrantees. Then, within each county using buyouts, the same socioeconomic and demographic characteristics are evaluated at a finer scale to describe the neighborhoods where properties were acquired. We used ZIP Code Tabulation Area (ZCTA) as a proxy for neighborhood, because this was the smallest scale possible based on FEMA’s public reporting of buyout locations by ZIP code.

**Fig. 5:**
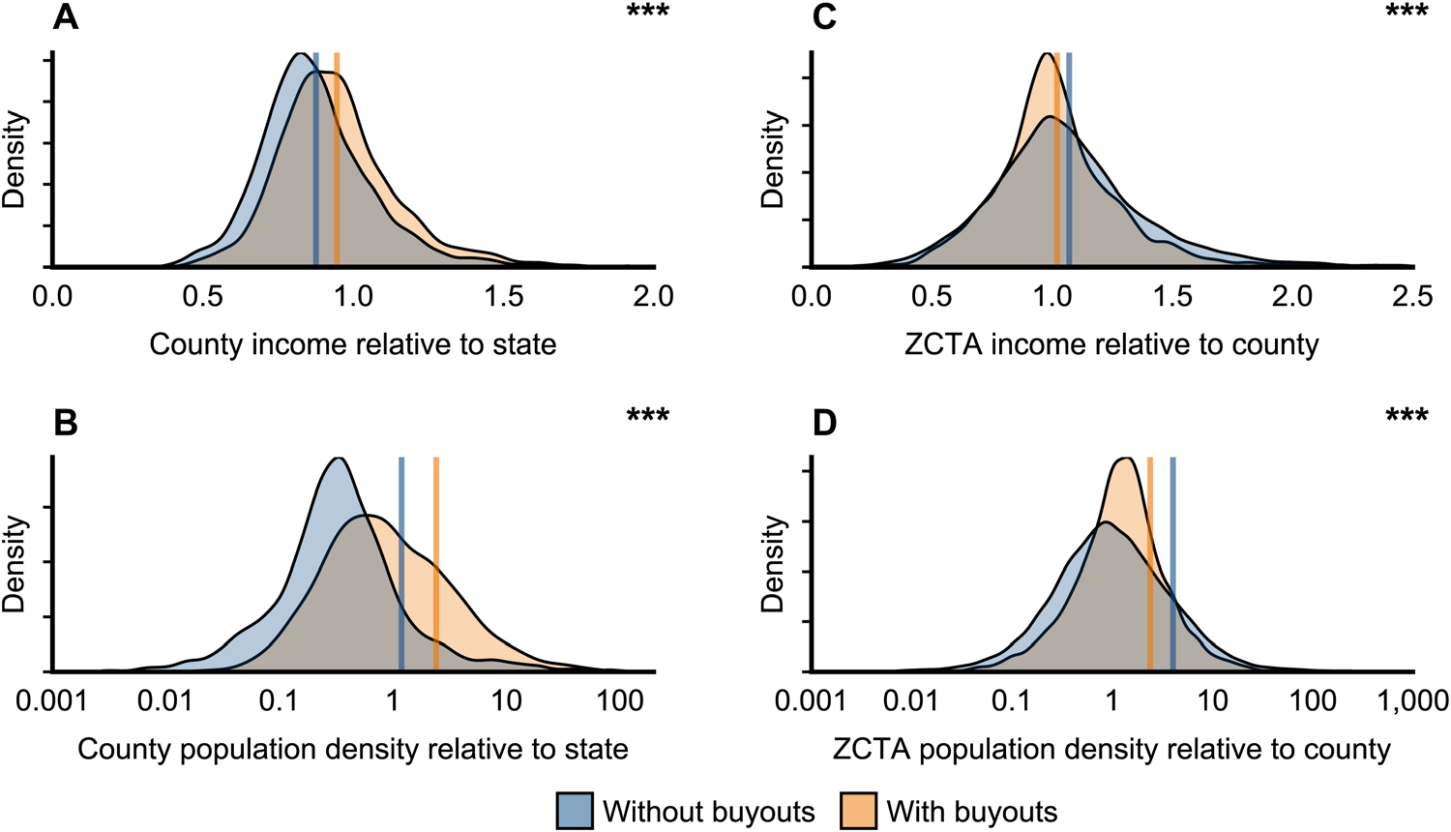
Socioeconomics and demographics of communities and residents participating in buyout programs, evaluating counties in which local governments have administered buyouts of flood-prone properties. In these 1087 counties, city or county governments have served as FEMA subgrantees over program years 1989–2017. These counties with local government–administered buyouts are compared to the 2019 counties in which no buyouts have occurred. (**A** and **B**) Density plots and means for counties with (orange) and without (blue) local government–administered buyouts for indicators averaged over 2012–2016: (A) median household income in county, divided by median household income in state, and (B) county population density, divided by state population density. (**C** and **D**) Within counties in which local governments have administered buyouts, density plots and means for ZCTAs in which property buyouts did (orange) and did not (blue) occur (16,718 ZCTAs in total: 2807 with buyouts and 13,911 without buyouts). In these panels, relative income and population density indicators compare ZCTAs to their corresponding counties. ***P ≤ 0.001 for Welch’s unequal variances *t* test for differences in means. Each panel includes data for the continental United States, Alaska, and Hawaii. Note that the *x* axis in some panels is truncated; data included in means and *t* tests, but not visualized graphically, are as follows: (A) 2 counties, (C) 65 ZCTAs, and (D) 179 ZCTAs.

Counties that have had locally administered buyout projects have higher income, education, population, and population density compared to counties without buyouts (Fig. 5 and fig. S7). However, residents in the ZCTAs in which buyouts occurred have lower income and population density than residents in other ZCTAs in the same county (Fig. 5 and figs. S8 and S9). ZCTAs with buyouts also have relatively lower education levels, lower English language proficiency, and greater racial diversity (fig. S8). For all indicators evaluated, the distributions for counties and ZCTAs with and without buyouts overlap, while the mean values characterized above differ with statistical significance (Fig. 5 and figs. S7 and S8). Note that, beyond the relative indicators of Fig. 5, data in figs. S7 and S8 additionally include socioeconomic and demographic indicators in absolute terms.

Implementation of FEMA-funded buyout projects is slow (fig. S10), with implications for implementing jurisdictions and affected neighborhoods and residents. The average FEMA HMGP buyout project takes 5.7 years from the start of the associated disaster event to project closeout (range, 0.4 to 16.8 years; median, 5.3 years) (fig. S10). Projects that purchase only one property are slightly shorter: 5.1 years on average (fig. S10). By the time a project is closed, properties have been purchased, structure demolitions or relocations have occurred, and the land has started to be maintained as open space. As an important note, the duration from a disaster event to project closeout imperfectly proxies key time frames for subgrantees and property owners. For example, deed transfer could occur well before closeout for the associated FEMA grant.

### Importance of flood exposure, socioeconomics, and demographics

**Fig. 6:**
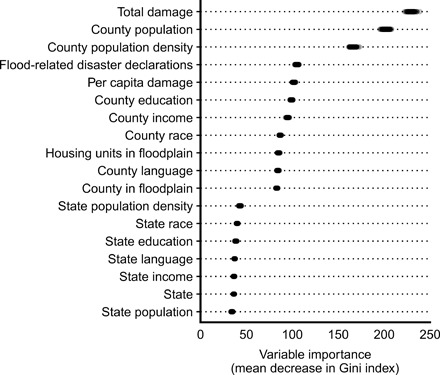
Variable importance in random forest model classifying counties with and without buyouts. Variables included encompass all factor categories assessed throughout this study: county-level exposure to flood-related hazards and county-level and state-level socioeconomic and demographic indicators. Results are shown for 100 model iterations, each based on 1000 decision trees. Mean decrease in Gini index indicates the importance of the variable in classifying counties as buyout or non-buyout counties. The 3108 counties included are those for which all assessed variables are available: 1115 with buyouts and 1993 without buyouts.

Integrating the above analyses, we use a random forest decision tree model to assess the comparative importance of different factors in distinguishing between counties in which buyouts have versus have not occurred (Fig. 6). This analysis considers all variable categories from above: county-level exposure to flood-related hazards, as well as county-level and state-level socioeconomic and demographic indicators. Across 100 random forest models (each based on 1000 decision trees), three variables emerge as most important in distinguishing counties with buyouts: total cumulative flood-related property damage over 1989–2016, county population, and county population density (Fig. 6). Classification error is 23% overall, 40% for buyout counties, and 13% for non-buyout counties.

## DISCUSSION

Voluntary property buyouts in the United States are among the longest-running programs of managed retreat globally, and they are the predominant means through which managed retreat has occurred in the United States to date (*12*). Increasingly, there is agreement that retreat from some areas will become an unavoidable option under intensifying climate change (*5*). Growing awareness of retreat has included analysis of ways to manage the process, along with coverage of the issue in the press, attention in the U.S. Congress, state and national programs supporting community relocation (e.g., LA SAFE, New Jersey Blue Acres), and boundary organizations dedicated to the topic (e.g., Climigration Network). The seeming inevitability of retreat has shifted emphasis to where and how it might occur, in light of challenges for implementing communities, individuals who relocate, and communities affected by the moves.

For FEMA-funded voluntary property buyouts in the United States to date, we use empirical analyses at a nationwide programmatic level to test hypotheses derived from case-based buyout studies and other managed retreat literature. Our empirical overview contradicts existing hypotheses in some cases, raises questions for future research, and notes areas for potential learning and improvement relevant as retreat increasingly occurs in response to climate change. These lessons can also inform future buyouts administered by local governments, as well as implementation through other policies and practices beyond historical experiences.

### Where buyouts have occurred

Economic analyses of flood risk management under increasing sea-level rise have suggested bifurcating coastal futures (*6*, *7*). These scenario-based evaluations identify responses that minimize the total cost of sea-level rise and imply that wealthier, more densely populated urban areas should increasingly engineer their coastlines with higher and stronger defensive structures. In poorer and more rural areas, however, it is not economically optimal to invest in protection, and these populations are expected to eventually retreat to higher ground.

By contrast, for government-funded retreat in the form of buyouts, our results indicate that richer, more densely populated areas have been more likely to implement voluntary buyouts of flood-prone properties to date. This finding does not imply that individual buyouts are not worthwhile investments; parcel-scale analyses show benefit-cost ratios far exceeding one. Nonetheless, the opposite pattern does bear multiple implications for future deployment of managed retreat, given the contrast between observed retreat and expectations based on economic efficiency and robustness. First, the finding may be indicative of the substantial human, financial, and other capacity required for a local government to implement a buyout, for example, to navigate the FEMA grant application process, procure additional funds, administer the process, and relocate participating property owners and residents (*8*, *14*, *19*). Other determinants of capacity, such as the presence of city planners or resilience officers, and also perceptions of climate risk and associated political will may be key enablers (*19*, *25*, *29*). Second, buyouts create many benefits that are not easily quantifiable in monetary terms. The open space resulting from buyouts may carry greater benefits in more urban areas with greater land competition by, for example, enabling recreation, nature conservation, or community revitalization in addition to flood mitigation (*14*, *29*–*31*). In understanding the benefits of retreat, multimetric consideration in monetary and broader terms may be important in understanding outcomes, including in urban areas with interacting drivers and land uses. Third, our finding of numerous early deployments of buyouts in the U.S. Midwest and more recent uptick of buyouts in the Northeast (Fig. 3B) may be indicative of growing demand for buyouts in coastal regions with high amenity and increased perception of risks from sea-level rise and coastal flooding (*32*–*34*). Fourth, if poor and rural coastal areas are forced to retreat under additional sea-level rise, as predicted in economic analyses, then our finding that greater government intervention has, to date, occurred in richer and more densely populated areas suggests that, without adjustments, future retreat in poorer and more rural communities may be less likely to be supported and managed by government. These populations could therefore be at increased risk of becoming trapped in areas of high flood risk (*35*, *36*).

### Issues of equity

Within counties with buyouts, however, the bought-out properties are located in relatively poorer, less densely populated areas, also with relatively lower education levels, lower English language proficiency, and greater racial diversity (fig. S8). Our analyses at the ZIP code level are inexact, limited by the spatial detail in publicly available FEMA records. Nonetheless, this pattern of acquisition in poorer, less dense, more marginalized areas (Fig. 5), which holds for inland and coastal shoreline counties evaluated separately, underscores the importance of ensuring socially just buyout policies and practices. Our analyses do not and cannot indicate whether this pattern is a result of programmatic design or purposeful intent to assist low-income areas. Nor can we determine whether white residents are relocating away from areas of racial diversity or whether people of color are relocated, as the FEMA data provide no indication of homeowner identity. Poorer, more marginalized people may be more likely to meet the eligibility requirements for buyouts (*11*, *37*), and they could potentially have more to gain from a move to opportunity (*38*). Alternatively, buyouts could be enabling white residents to relocate away from areas of greater racial diversity. Relevant to these alternatives, previous case-based analyses have suggested that, when social equity is not explicit, inequitable implementation practices or outcomes may occur (*11*, *21*, *28*, *39*). These dynamics can include perceived coercion (*8*, *27*), local-level political pressures favoring flood hazard mitigation for the privileged over the marginalized (*26*), more deliberate findings of substantial damage in socially vulnerable areas, or relocations to areas with equal flood risk and greater social vulnerability (*29*). Our crosscutting finding similarly points to the importance of evaluating the equity of buyout implementation and outcomes to date.

### Small deployments to date

Perhaps surprisingly, FEMA-funded projects supporting buyouts have frequently been small, only one to three bought-out properties each (Fig. 3A and fig. S5). Moreover, most counties have used buyouts only once or twice. Counties with buyouts have implemented a median of two such FEMA-funded grants. Buyout projects that acquire only a few properties effectively increase the administrative burden per bought-out property for participating communities, also affecting the economic efficiency of investments. Encouragingly, buyouts are more likely in counties with greater exposure to flood-related hazards (Fig. 4). But small buyout projects may result in a sparse or patchy removal of properties and may miss opportunities to more strategically restore floodplains and reduce overall flood risk within communities (*17*), reducing cost-effectiveness of projects. We do not know whether use of buyouts has been small on purpose, perhaps to help a handful of disaster survivors who wish to relocate or to assist residents with political or financial power (*29*, *40*), or by accident, perhaps because of low participation rates (*13*, *41*). This remains an open question for future research. However, while small numbers of buyouts have been the norm, the counties that have had many buyouts (e.g., the 100 counties with 100 or more bought-out properties) are more readily analogous to the type of large-scale retreat expected in response to climate change (*16*, *26*). Better understanding of why buyout projects and county-level deployments have been small to date may help future efforts to scale up.

### Areas relevant to policy improvement and learning

Our empirical analyses raise issues relevant to learning in the buyout policy process (*16*). First, in successive decades since 1989, projects have decreased, rather than increased, in size (fig. S5). This trend could result from programmatic or funding shifts affecting prioritization of buyouts within FEMA assistance for flood hazard mitigation or from failure to iteratively streamline and refine buyout policies (*16*). Identifying why project sizes have been shrinking might help reverse the trend to meet anticipated future needs.

Second, the implementation of buyout projects has been slow (fig. S10). The implications for participating communities, property owners, and residents merit further evaluation (*16*, *25*, *26*). We rely on data for overall buyout-project durations, although other shorter time frames are noteworthy. The shorter time frames include the time from a flood-related disaster to the specific transaction dates for property purchases and when homeowners can eliminate mortgage obligations (*40*). The date of property demolition is also meaningful, as it is when a neighborhood starts to have a vacant lot or restored open space (*14*).

Third, our study emphasizes the importance of increased transparency in FEMA reporting, enabling evaluation and associated policy learning through time (*11*, *16*). For example, in buyout data publicly posted by FEMA, more than 50% of entries are empty for some fields (e.g., Fig. 3D). Without full accurate data, programmatic evaluations are likely to be incomplete, affecting understanding of the economic efficiency and social equity of buyouts to date.

Throughout these analyses, we have relied on publicly available data, with their associated limitations. Despite these limitations, our crosscutting treatment provides a needed foundation for understanding trends and drivers where property buyouts have happened, and where they have not, in the United States to date. Beyond buyouts as historically practiced, future retreat may include experimentation with different practices and policies, including funding, facilitation, and coordination to different degrees. Across these approaches, both the challenges and lessons of buyouts can inform and support strategies of managed retreat across diverse contexts in response to climate change.

## MATERIALS AND METHODS

### Study design

The aim of this study was an analysis of all FEMA-funded voluntary buyouts of flood-prone properties. We built upon hypotheses generated from case-based research to identify broader patterns regarding where and how buyouts occur. Attuned to effectiveness and equity of managed retreat in the United States to date, our assessment determined (i) where voluntary buyouts of flood-prone properties have taken place, (ii) what flood-related hazards and damage have occurred in areas with and without buyouts, and (iii) what socioeconomic and demographic characteristics predict which jurisdictions have implemented buyouts and which neighborhoods were bought out. For objective (i), our empirical treatment evaluated all buyouts for which information was publicly available. For objectives (ii) and (iii), we analyzed available indicators of exposure to flood-related hazards and of socioeconomics and demographics. We applied each indicator over the full geographic scope for which it was available, and in Results, we report on all analyses completed.

### FEMA-funded voluntary buyouts of flood-prone properties

In the United States, government acquisition of flood-prone properties has generally been funded by federal agencies and administered by state or local governments (Fig. 1). The largest and longest-running program is FEMA’s HMGP, which started in 1989 and expanded after the 1993 Great Midwest Floods. FEMA has also funded property acquisitions through other initiatives such as the Flood Mitigation Assistance Grant Program (FMA), the Pre-Disaster Mitigation Grant Program (PDM), the Repetitive Flood Claims Grant Program (RFC), and the Severe Repetitive Loss Grant Program (SRL). Under FEMA regulations, acquisitions of flood-prone properties must be voluntary, which means property owners agree to sell their properties, and state and local officials cannot use eminent domain or condemnation powers. Applicable flood-related hazards include flows or accumulation of water, flows of mud, and, in some cases, erosion, and we adopt this broad definition in analysis of flood-related hazards and flood-prone properties. HMGP-supported acquisitions occur after a federal declaration of major disaster.

Data on FEMA-funded voluntary buyouts of flood-prone properties, through all of these programs, were accessed in April 2018 from the OpenFEMA dataset of hazard mitigation assistance mitigated properties (https://www.fema.gov/openfema-dataset-hazard-mitigation-assistance-mitigated-properties-v1). On the basis of the “property action,” “title,” and “type” for each entry, the dataset was manually cleaned to identify 43,633 entries corresponding, with high likelihood, to voluntary property buyouts. In most cases, the entries were voluntary buyouts in which a property was acquired and then demolished, with the land subsequently maintained as open space. In a small number of cases (~1% of entries), the entries were voluntary buyouts in which a property was relocated rather than demolished, yet still with the land subsequently maintained as open space.

A buyout project is defined in this study as a FEMA grant (Fig. 1) that involves at least one bought-out property and may also include other property buyouts or additional hazard mitigation measures. Program year is, for HMGP projects, the fiscal year of the associated disaster declaration. For other programs, program year is simply the fiscal year of the associated project. Date closed is the date by which project closeout has occurred. At project closeout, all applicable administrative actions and required work of the award have been completed (https://www.fema.gov/hmgp-appeal-keywords/9119). For buyout projects, subgrantee information was accessed in May 2018 from the OpenFEMA dataset of hazard mitigation assistance projects (https://www.fema.gov/openfema-dataset-hazard-mitigation-assistance-projects-v1).

### Flood-related federal disaster declarations

Major disasters declared were accessed in July 2018 from the OpenFEMA dataset of disaster declarations summaries (https://www.fema.gov/openfema-dataset-disaster-declarations-summaries-v1). Flood-related major disaster declarations were defined as those containing “flood,” “hurricane,” “storm,” “coast,” or “tsunami” within the title or incident type of the disaster. Of 1462 major disaster declarations over 1989–2017, 1376 were designated as flood related on this basis. For the flood-related declarations of major disasters that involved individual assistance, the declarations have the following incident types: severe storm(s) (400 declarations), flood (134), hurricane (106), tornado (34), coastal storm (8), severe ice storm (7), fire (4), snow (1), and mud/landslide (1). Most counties across the United States experienced major disaster declarations over 1989–2017: Of 3220 counties and county-equivalent entities in U.S. states and Puerto Rico, only 73 had no major disaster declarations of any type, only 115 had no flood-related major disaster declarations, and only 254 had no flood-related major disaster declarations involving individual assistance.

### Flood hazard maps

Areas with flood hazard were evaluated based on the estimates of Wing *et al*. (*4*, *23*). These estimates include riverine and rainfall-driven flooding. We applied their maps of areas with flood events that have a 1% chance of being equaled or exceeded in any given year (i.e., 1% annual exceedance probability or 100-year flood zones). Compared to FEMA Special Flood Hazard Areas, these flood zones are based on higher-resolution, more accurate terrain data, and improved fluid physics, and they incorporate known flood defenses and all river catchment scales (*4*, *23*). Wing *et al*. (*4*) estimate that approximately 41 million Americans live within 1% annual chance floodplain, as compared to approximately 13 million Americans based on FEMA’s flood maps.

### Flood-related property damage

Flood-related property damage data were obtained from the SHELDUS version 16.1 database (*42*). Flood-related perils were used to determine flood-related property damage. Data evaluated include property damage in inflation-adjusted U.S. dollars as well as property damage per capita. The perils correspond to the following hazard types: hurricane/tropical storm (62.0% of total cumulative damage in the United States over 1989–2016), flooding (32.6%), severe storm/thunderstorm (2.7%), winter weather (0.9%), coastal (0.7%), wind (0.5%), hail (0.3%), tornado (0.2%), landslide (<0.1%), tsunami/seiche (<0.1%), lightning (<0.1%), and fog (<0.1%).

### Socioeconomic and demographic indicators

A variety of commonly applied indicators were used to proxy capacity and social vulnerability of implementing communities and bought-out residents (including property owners and tenants) participating in FEMA-funded programs for voluntary buyouts of flood-prone properties. Socioeconomic and demographic data analyzed originate from the U.S. Census American Community Survey (https://www.census.gov/programs-surveys/acs). From the American Community Survey, 5-year estimates over 2012–2016 were used for the following variables: income, education, racial diversity, population, population density, and household English language proficiency (operational definitions provided in captions for Fig. 5 and figs. S7 and S8) (*24*).

For buyouts administered by local government subgrantees (i.e., city or county governments), county-scale socioeconomic and demographic indicators were used to evaluate the capacity of the local government to apply for, receive, and administer federal funding. County-scale data could be systematically analyzed for all local government subgrantees, which is why this scale of analysis was applied. County-scale socioeconomic and demographic indicators were evaluated in absolute terms and also in relative terms, comparing each county to its respective state.

Within counties where local government has administered one or more buyout projects, the same socioeconomic and demographic characteristics were also evaluated at a finer ZCTA scale. This scale of analysis was applied to proxy the capacity and social vulnerability of neighborhoods and residents participating in buyout programs. ZCTA-scale indicators were similarly evaluated in absolute terms and also in relative terms, here comparing each ZCTA to the respective county.

### Statistical analysis

Data were analyzed in R 3.5.1 (RStudio 1.1.463), ArcGIS 10.5.1 ArcMap, and Microsoft Excel. Statistical analyses applied include Welch’s unequal variances *t* test for differences in means, Kruskal-Wallis one-way analysis of variance, spatial statistics (e.g., zonal statistics in ArcMap), and random forest classification analysis. All specified type I error rates in this article apply to only the particular test in question and not the series of tests.

The random forest analysis, which is a decision tree method, was performed with the randomForest package in R (*43*). This analysis was used to determine variable importance in classifying counties in which buyouts have versus have not occurred. The following variables at the county level were included: 5-year American Community Survey census data for income, education, racial diversity, population, population density, and household English language proficiency; flood-related federal disaster declarations from 1989 to 2017; cumulative flood-related property damage per capita and in total over 1989–2016; and fraction of county area and of county housing units in 1% annual chance floodplain. Variables for state-level census data and state identity were also included. A random forest model is an ensemble of multiple decision trees, each of which is fit on a different subset of data. In our analysis, each random forest consisted of 1000 decision trees. The importance of variables for classification was calculated through mean decrease in Gini index for each variable.

## Supplementary Material

http://advances.sciencemag.org/cgi/content/full/5/10/eaax8995/DC1

Download PDF

Managed retreat through voluntary buyouts of flood-prone properties

## SUPPLEMENTARY MATERIALS

Supplementary material for this article is available at http://advances.sciencemag.org/cgi/content/full/5/10/eaax8995/DC1 Supplementary Material Fig. S1. Flood-related property damage in the continental United States, Alaska, and Hawaii. Fig. S2. Spatial patterns of flood-related federal disaster declarations over 1989–2017. Fig. S3. Spatial and temporal trends in flood-related property damage over 1989–2016. Fig. S4. FEMA-funded buyouts of flood-prone properties over program years 1989–2017, by grant program. Fig. S5. The frequency of buyout projects (no. of projects) of different sizes (no. of bought-out properties) for overall program years 1989–2017 and for specific decades 1989–1998, 1999–2008, and 2009–2017. Fig. S6. Flood-related exposure in counties in which voluntary property buyouts have and have not occurred. Fig. S7. Socioeconomics and demographics of communities participating in buyout programs, evaluating counties in which local governments have administered buyouts of flood-prone properties. Fig. S8. Socioeconomics and demographics of residents participating in buyout programs. Fig. S9. Population and population density within counties with local government–administered buyouts. Fig. S10. The duration of FEMA HMGP projects with property buyouts over program years 1989–2017.
