# Supplementary material for: Managed retreat through voluntary buyouts of flood-prone properties
Source: Sci Adv. 2019 Oct 9;5(10):eaax8995. doi: 10.1126/sciadv.aax8995 (PMC6785245; doi:10.1126/sciadv.aax8995)
Supplement: http://advances.sciencemag.org/cgi/content/full/5/10/eaax8995/DC1 [file supp_5_10_eaax8995__index.html]

Science Advances | Science AdvancesAAASSearchScience AdvancesMenu

## Supplementary Materials

**This PDF file includes:**

- Supplementary Material
- Fig. S1. Flood-related property damage in the continental United States, Alaska, and Hawaii.
- Fig. S2. Spatial patterns of flood-related federal disaster declarations over 1989–2017.
- Fig. S3. Spatial and temporal trends in flood-related property damage over 1989–2016.
- Fig. S4. FEMA-funded buyouts of flood-prone properties over program years 1989–2017, by grant program.
- Fig. S5. The frequency of buyout projects (no. of projects) of different sizes (no. of bought-out properties) for overall program years 1989–2017 and for specific decades 1989–1998, 1999–2008, and 2009–2017.
- Fig. S6. Flood-related exposure in counties in which voluntary property buyouts have and have not occurred.
- Fig. S7. Socioeconomics and demographics of communities participating in buyout programs, evaluating counties in which local governments have administered buyouts of flood-prone properties.
- Fig. S8. Socioeconomics and demographics of residents participating in buyout programs.
- Fig. S9. Population and population density within counties with local government–administered buyouts.
- Fig. S10. The duration of FEMA HMGP projects with property buyouts over program years 1989–2017.

Download PDF

**Files in this Data Supplement:**

- Adobe PDF - aax8995\_SM.pdf
