# Supplementary material for: Managed retreat through voluntary buyouts of flood-prone properties
Source: Sci Adv. 2019 Oct 9;5(10):eaax8995. doi: 10.1126/sciadv.aax8995 (PMC6785245; doi:10.1126/sciadv.aax8995)
Supplement: Download PDF [file aax8995_SM.pdf]

Science Advances
AAAS

advances.sciencemag.org/cgi/content/full/5/10/eaax8995/DC1

Supplementary Materials for

Managed retreat through voluntary buyouts of flood-prone properties

Katharine J. Mach*, Caroline M. Kraan, Miyuki Hino, A. R. Siders, Erica M. Johnston, Christopher B. Field

*Corresponding author. Email: kmach@rsmas.miami.edu

Published 9 October 2019, *Sci. Adv.* **5**, eaax8995 (2019)
DOI: 10.1126/sciadv.aax8995

This PDF file includes:

Supplementary Material
Fig. S1. Flood-related property damage in the continental United States, Alaska, and Hawaii.
Fig. S2. Spatial patterns of flood-related federal disaster declarations over 1989–2017.
Fig. S3. Spatial and temporal trends in flood-related property damage over 1989–2016.
Fig. S4. FEMA-funded buyouts of flood-prone properties over program years 1989–2017, by grant program.
Fig. S5. The frequency of buyout projects (no. of projects) of different sizes (no. of bought-out properties) for overall program years 1989–2017 and for specific decades 1989–1998, 1999–2008, and 2009–2017.
Fig. S6. Flood-related exposure in counties in which voluntary property buyouts have and have not occurred.
Fig. S7. Socioeconomics and demographics of communities participating in buyout programs, evaluating counties in which local governments have administered buyouts of flood-prone properties.
Fig. S8. Socioeconomics and demographics of residents participating in buyout programs.
Fig. S9. Population and population density within counties with local government–administered buyouts.
Fig. S10. The duration of FEMA HMGP projects with property buyouts over program years 1989–2017.

SUPPLEMENTARY MATERIAL

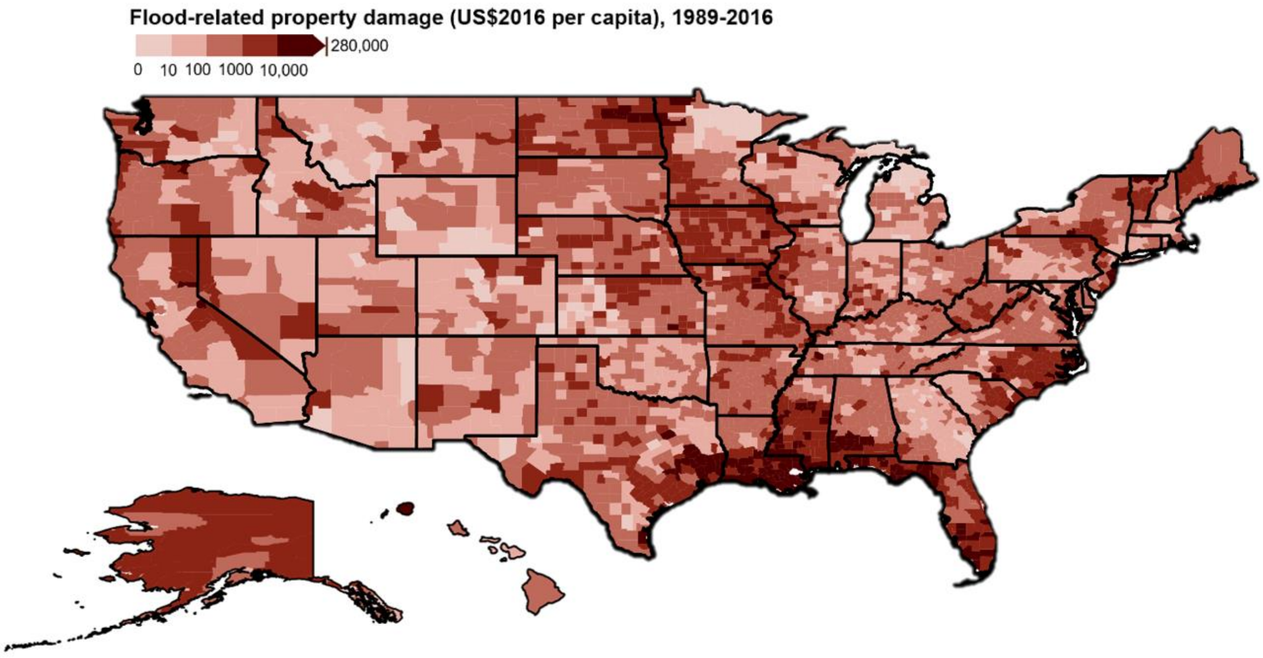

Fig. S1. Flood-related property damage in the continental United States, Alaska, and Hawaii. For each county, cumulative damage over 1989–2016 is depicted (as US$2016 per capita). Compared to Fig. 2B, this figure shows per-capita, rather than total, cumulative damage. These SHELDUS property-damage estimates are not available for Puerto Rico.

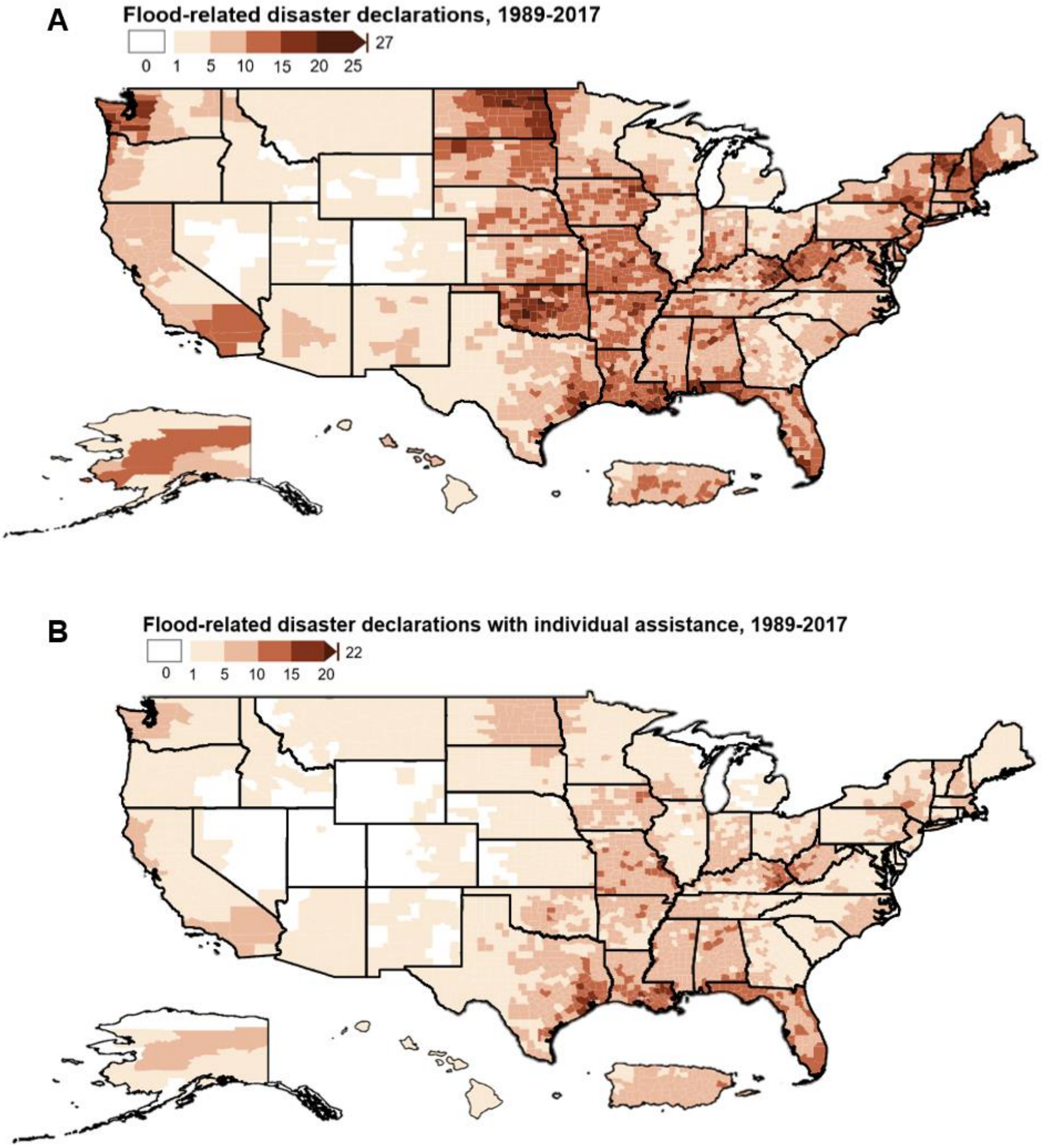

Fig. S2. Spatial patterns of flood-related federal disaster declarations over 1989–2017. The number of major disasters declared is shown (**A**) in counties that received individual or public assistance and (**B**) only in counties that received individual assistance.

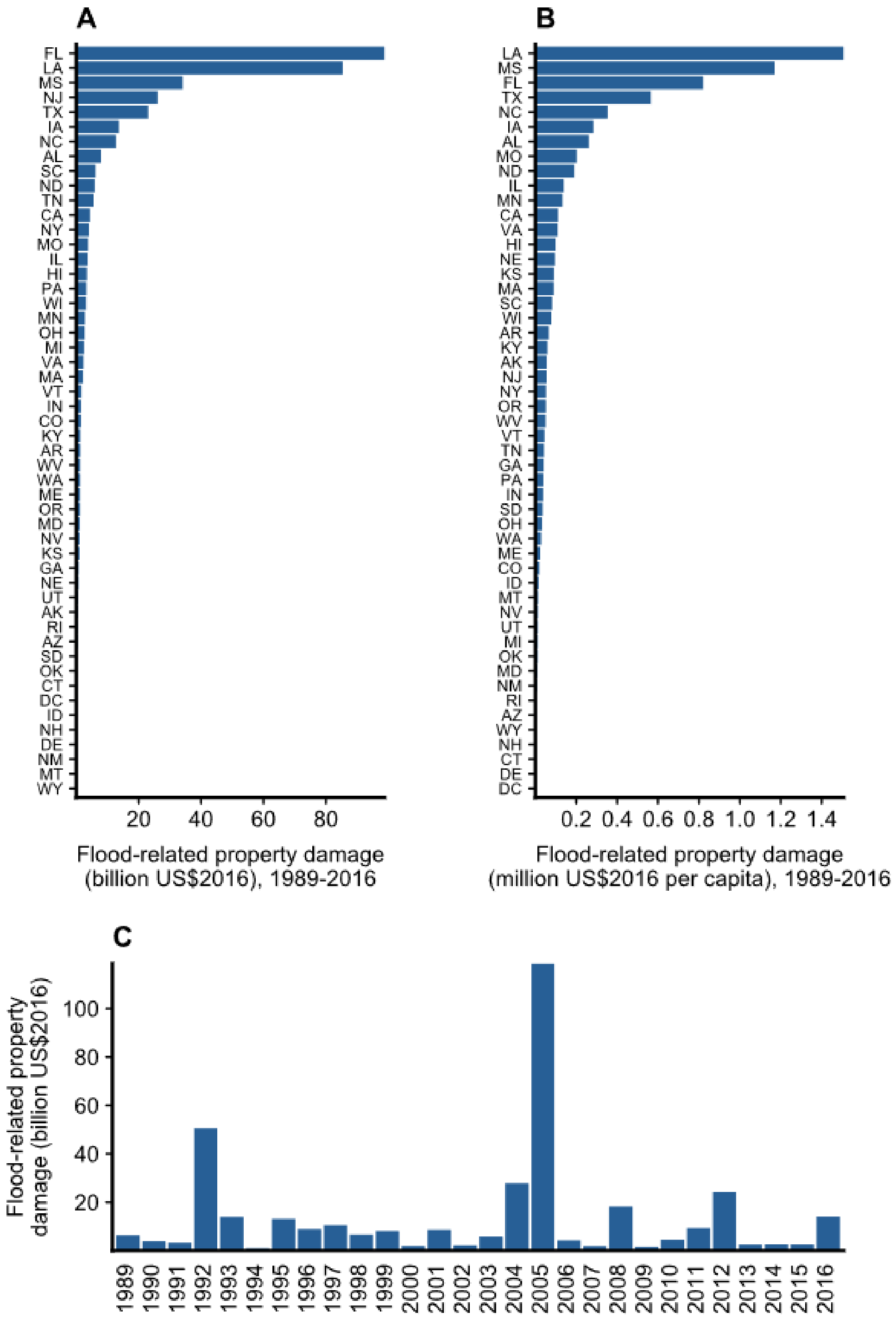

Fig. S3. Spatial and temporal trends in flood-related property damage over 1989–2016. (**A**) Total cumulative damage for each state (as billion US$2016). (**B**) Per-capita cumulative damage for each state (as million US$2016 per capita). (**C**) Total cumulative damage in each year (as billion US$2016).

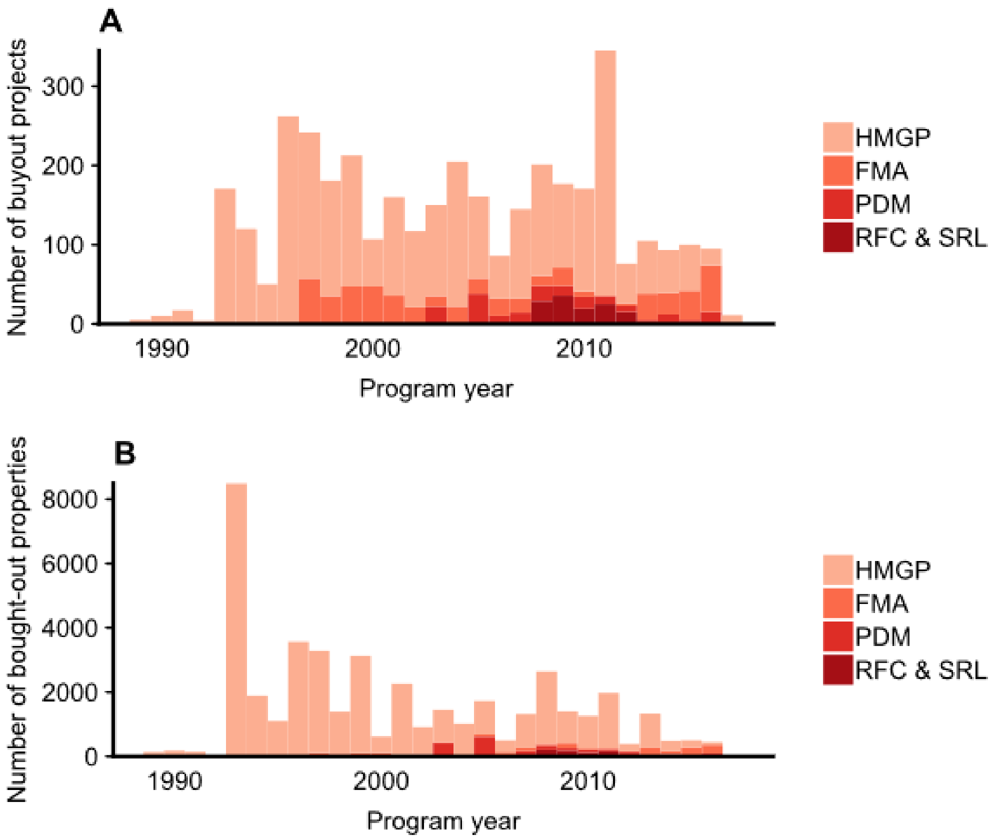

Fig. S4. FEMA-funded buyouts of flood-prone properties over program years 1989–2017, by grant program. (**A**) The number of buyout projects through time across the FEMA grant programs supporting voluntary buyouts. Each project included here involves at least one bought-out property and may also include other property buyouts or additional hazard mitigation measures. (**B**) For projects under each grant program, the total number of bought-out properties each program year.

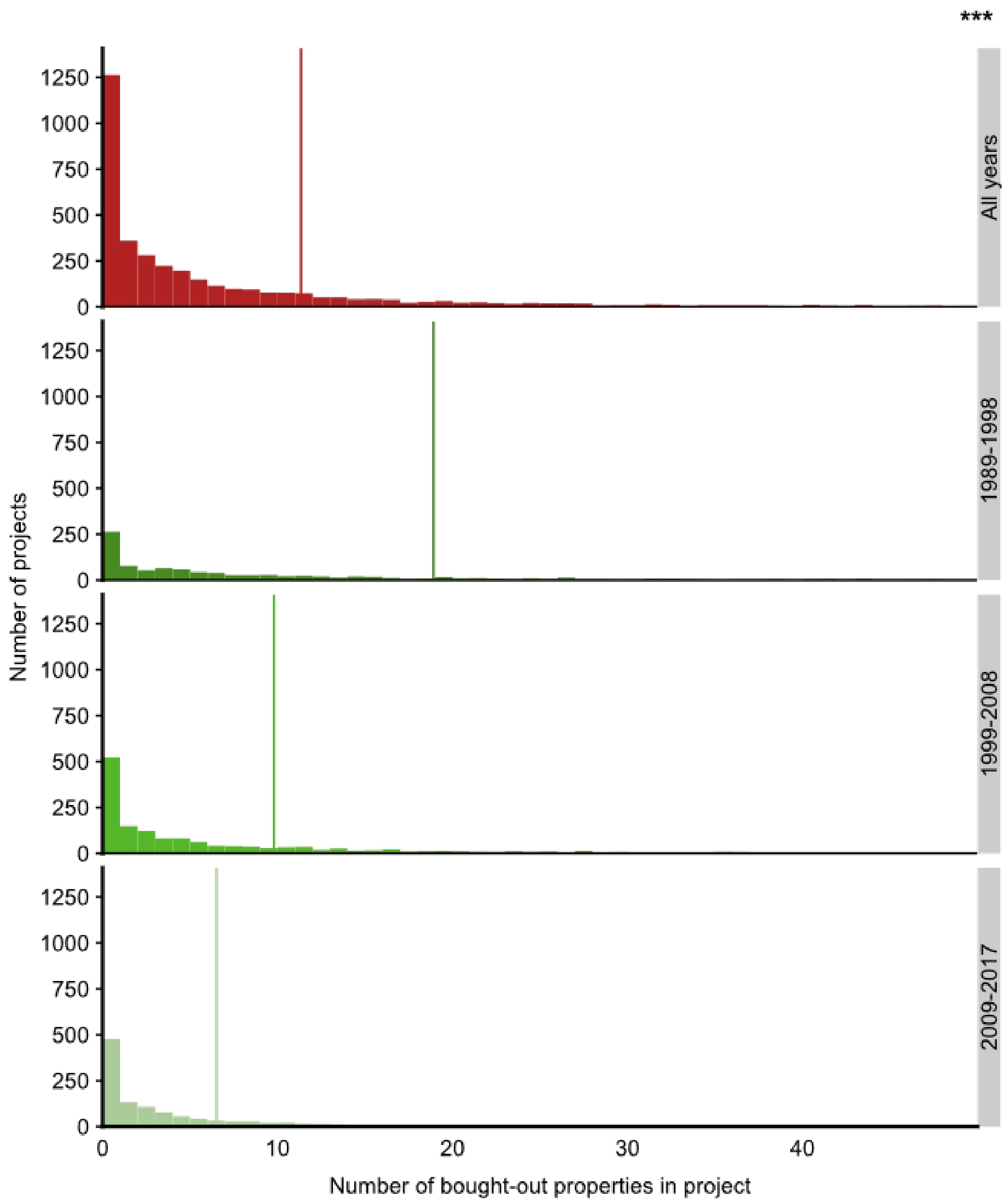

Fig. S5. The frequency of buyout projects (no. of projects) of different sizes (no. of bought-out properties) for overall program years 1989–2017 and for specific decades 1989–1998, 1999–2008, and 2009–2017. In each panel, mean project size is specified with a vertical line. The mean size of buyout projects (as no. of bought-out properties), as well as the maximum size, declines in successive decades. *** indicates $p \leq 0.001$ for Kruskal–Wallis one-way analysis of variance across the decades, with pairwise comparisons all statistically significant at the same level. Note that the x-axis in each panel is truncated; 143 out of the 3780 buyout projects overall (3.8%) involve more than 50 bought-out properties. These projects, though not visualized graphically, are included in mean project sizes specified and the statistical tests.

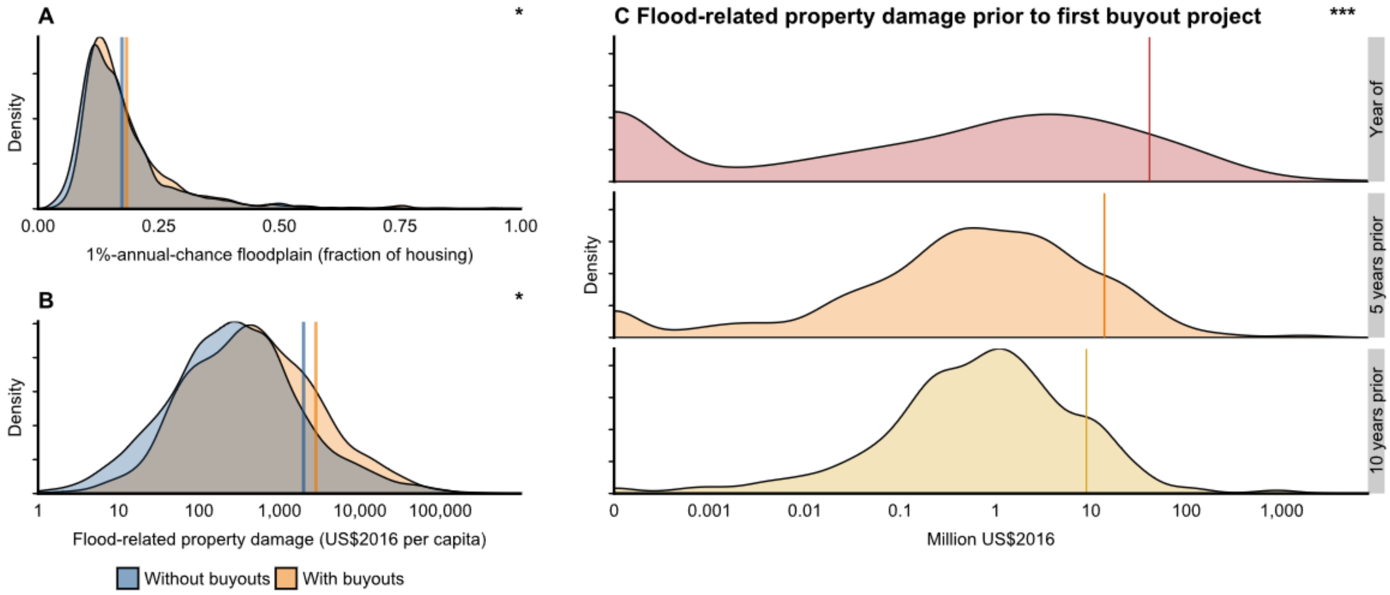

Fig. S6. Flood-related exposure in counties in which voluntary property buyouts have and have not occurred. (**A** and **B**) Flood-related exposure in counties in which voluntary property buyouts have versus have *not* occurred, by (**A**) fraction of county housing units that are in 1%-annual-chance floodplain and (**B**) cumulative flood-related property damage over 1989–2016 (as US$2016 per capita). Within each panel, the density plot and mean are orange for counties in which voluntary property buyouts have been administered by any subgrantees over program years 1989–2017, whereas they are blue for counties in which buyouts have *not* occurred. * indicates $0.001<p\leq0.05$ for Welch's unequal variances t-test for differences in means. (**C**) Temporal dynamics of flood-related property damage in counties in which voluntary property buyouts have occurred. Flood-related property damage here is cumulative damage per year (as million US$2016). For the first buyout project in each county, density plots of counties are shown, by flood-related property damage in the program year of the buyout project and the five and 10 years prior (as mean yearly damage). *** indicates $p\leq0.001$ for Kruskal–Wallis one-way analysis of variance across the damage timeframes, with pairwise comparisons of year-of and prior damages statistically significant at the $0.001<p\leq0.05$ level. Data are included for all geographic regions available for each flood-related measure: (**A**–**C**) continental United States; (**B**, **C**) AK, HI. The number of counties relevant to each panel is therefore as follows: (**A**) 3108 counties (1993 in blue, without buyouts; 1115 in orange, with buyouts); (**B**) 3142 counties (2019 blue, 1123 orange); (**C**) 1123 counties with buyouts.

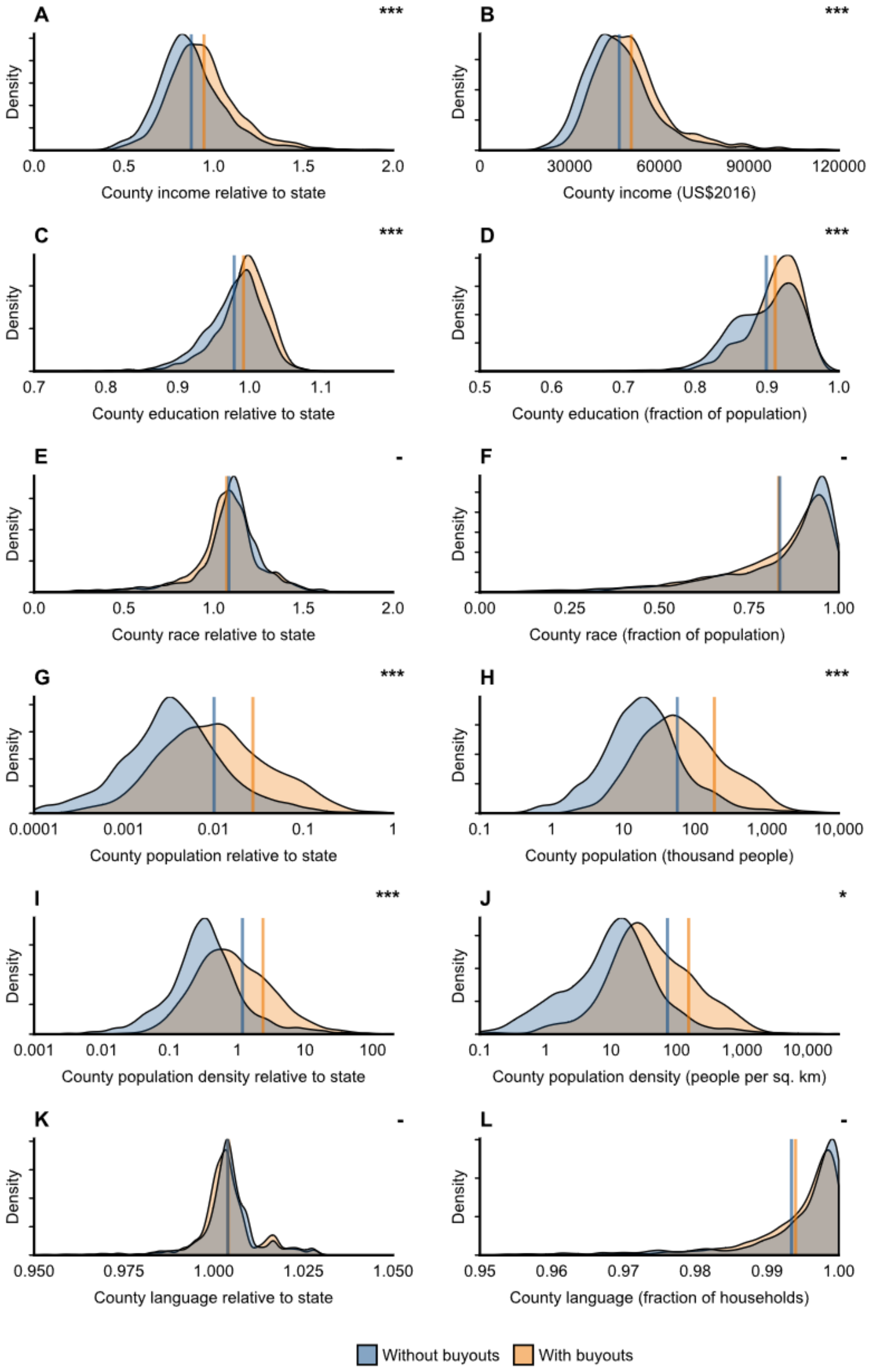

A ***
Density
0.0 0.5 1.0 1.5 2.0
County income relative to state
B ***
Density
0 30000 60000 90000 120000
County income (US$2016)
C ***
Density
0.7 0.8 0.9 1.0 1.1
County education relative to state
D ***
Density
0.5 0.6 0.7 0.8 0.9 1.0
County education (fraction of population)
E -
Density
0.0 0.5 1.0 1.5 2.0
County race relative to state
F -
Density
0.00 0.25 0.50 0.75 1.00
County race (fraction of population)
G ***
Density
0.0001 0.001 0.01 0.1 1
County population relative to state
H ***
Density
0.1 1 10 100 1,000 10,000
County population (thousand people)
I ***
Density
0.001 0.01 0.1 1 10 100
County population density relative to state
J *
Density
0.1 1 10 100 1,000 10,000
County population density (people per sq. km)
K -
Density
0.950 0.975 1.000 1.025 1.050
County language relative to state
L -
Density
0.95 0.96 0.97 0.98 0.99 1.00
County language (fraction of households)
Without buyouts
With buyouts

Fig. S7. Socioeconomics and demographics of communities participating in buyout programs, evaluating counties in which local governments have administered buyouts of flood-prone properties. In these 1087 counties, city or county governments have served as FEMA subgrantees over program years 1989–2017. These counties with local-government-administered buyouts are compared to the 2019 counties in which no buyouts have occurred. Density plots and means of counties with (orange) and without (blue) local-government-administered buyouts are shown for relative and absolute indicators averaged over 2012–2016: (**A**) relative income – median household income in county, divided by median household income in state. (**B**) absolute income – median household income (total household income over 12 months) in county (US$2016). (**C**) relative education – fraction of county population over 25 with high school diploma or equivalent credential, divided by fraction of state population over 25 with high school diploma or equivalent credential. (**D**) absolute education – fraction of county population over 25 with high school diploma or equivalent credential. (**E**) relative racial diversity – fraction of county population that is white only, divided by fraction of state population that is white only. (**F**) absolute racial diversity – fraction of county population that is white only. (**G**) relative population – county population, divided by state population. (**H**) absolute population – county population (thousands of people). (**I**) relative population density – county population density, divided by state population density. (**J**) absolute population density – county population density (number of people per square kilometer). (**K**) relative English language proficiency – fraction of county households not limited in speaking English, divided by fraction of state households not limited in speaking English. (**L**) absolute English language proficiency – fraction of county households not limited in speaking English (with limited English speaking household defined as a household with no member 14 years old or over speaking only English or speaking English very well). Significance levels are shown for Welch's unequal variances t-test for differences in means: *** indicates $p \leq 0.001$; * indicates $0.001 < p \leq 0.05$; - indicates $p > 0.05$. Each panel includes data for the continental United States, AK, and HI. Note that the x-axis in some panels is truncated; data included in means and t-tests, but not visualized graphically, are as follows: (**A**) 2 counties, (**B**) 1 county, (**G**) 27 counties, (**H**) 2 counties, (**J**) 8 counties, (**K**) 9 counties, (**L**) 29 counties.

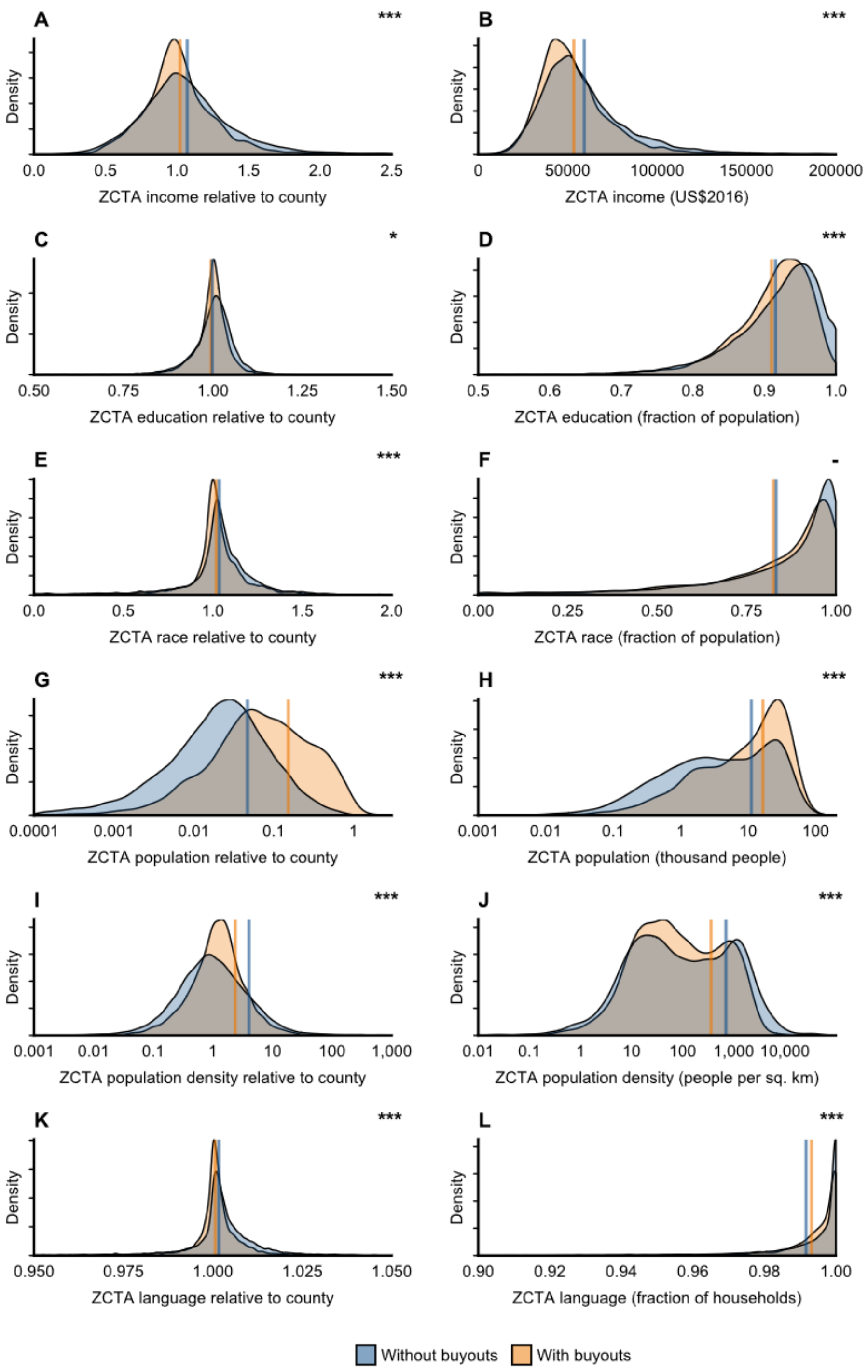

Fig. S8. Socioeconomics and demographics of residents participating in buyout programs. *Within* counties in which local governments have administered buyouts, density plots and means of ZCTAs in which property buyouts did (orange) and did not (blue) occur (16,718 ZCTAs in total: 2807 with buyouts, 13,911 without buyouts). In these panels, relative socioeconomic and demographic indicators are defined as in fig. S7, except here they compare ZCTAs to their corresponding counties; absolute indicators are also as in fig. S7, except here they are at ZCTA scale. Significance levels are shown for Welch's unequal variances t-test for differences in means: *** indicates $p \leq 0.001$; * indicates $0.001 < p \leq 0.05$; - indicates $p > 0.05$. Each panel includes data for the continental United States, AK, and HI. Note that the x-axis in some panels is truncated; data included in means and t-tests, but not visualized graphically, include 0–242 ZCTAs across panels.

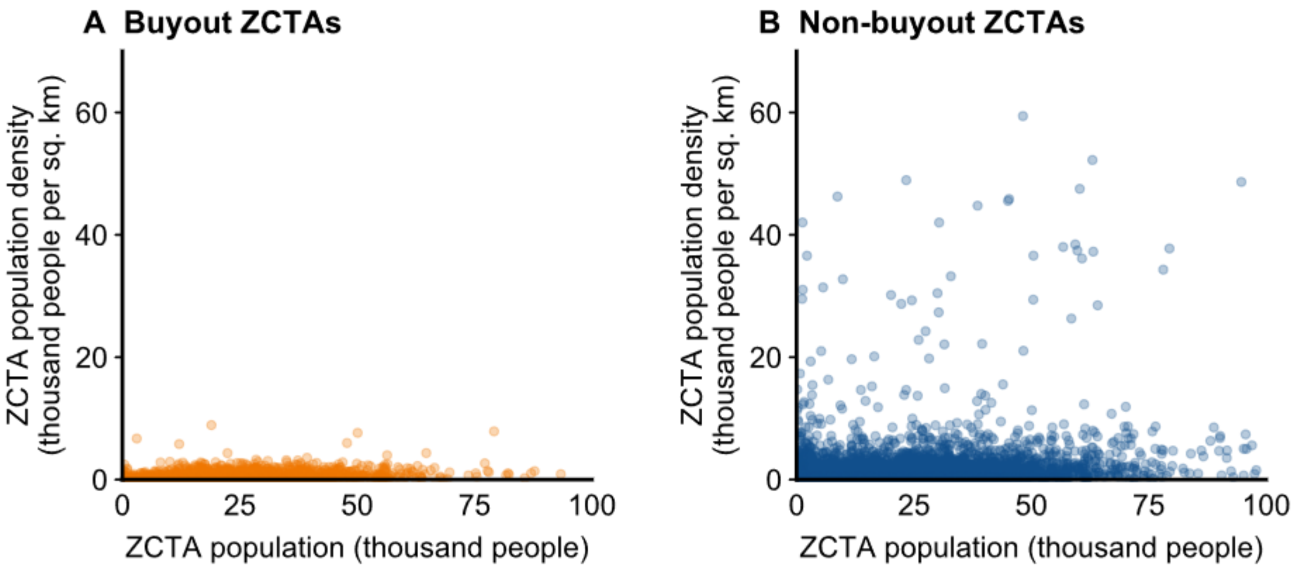

Fig. S9. Population and population density within counties with local government–administered buyouts. (**A**) Population and population density within ZCTAs in which property buyouts occurred (2807 ZCTAs in total). (**B**) Population and population density within ZCTAs in which property buyouts did *not* occur (13,911 ZCTAs in total). Eight ZCTAs with population greater than 100,000 people are not visualized in this figure, although they are included in associated statistical analyses in fig. S8.

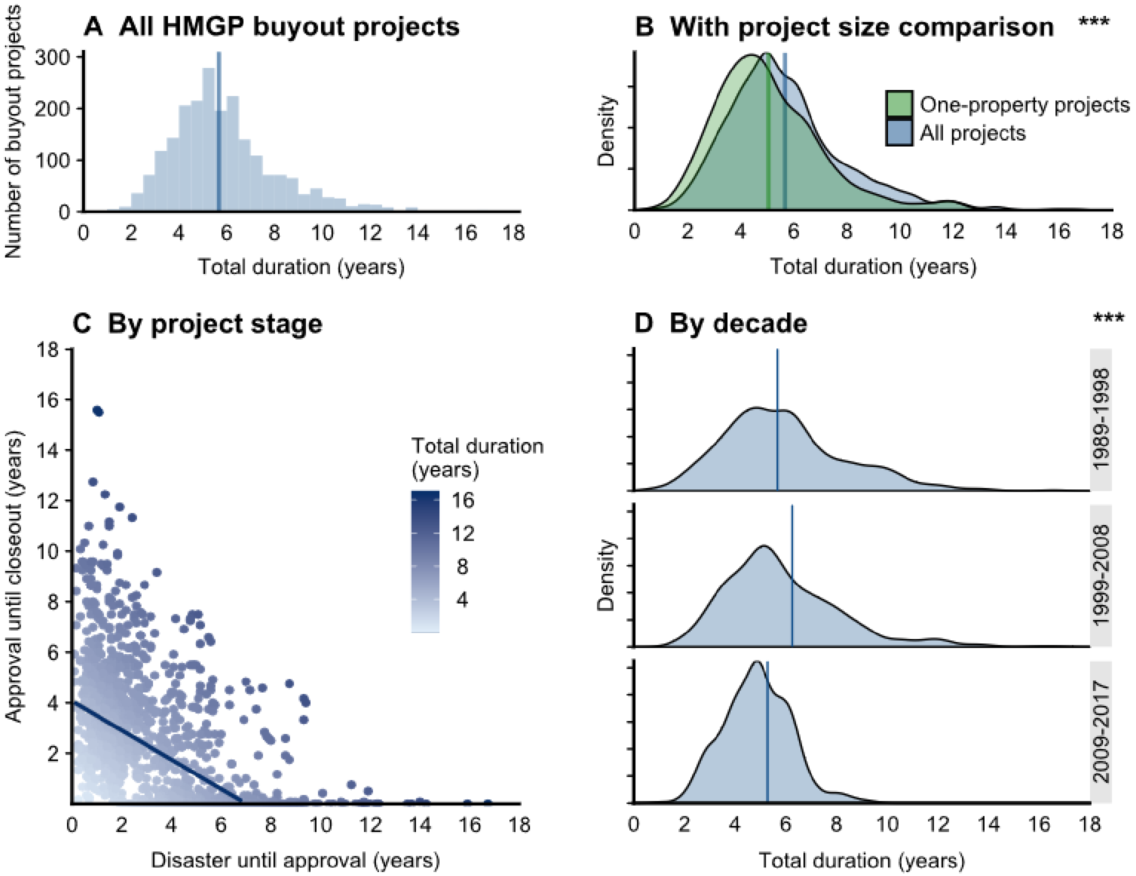

Fig. S10. The duration of FEMA HMGP projects with property buyouts over program years 1989–2017. (**A**) The frequency of buyout projects of different total durations (in 6-mo. bins). Mean project duration is specified with vertical line. Each project included here involves at least one bought-out property and may also include other property buyouts or additional hazard mitigation measures. The total duration of a project is defined as the time from the start of the associated disaster event (mo. of disaster) to project closeout (mo. of closeout). At project closeout, associated property acquisitions and structure demolitions or relocations have occurred, and the land has started to be maintained as open space. (**B**) Density plots of buyout projects by total project duration, again with mean project duration specified with vertical lines. The durations of all HMGP buyout projects (blue, 2157 projects) are compared to the durations of HMGP buyout projects including only a *single* bought-out property (that is, projects without additional property buyouts or other hazard mitigation measures; green, 521 projects). The one-property projects are shorter in duration (Welch's unequal variances t-test for differences in means, $p \leq 0.001$). (**C**) A comparison of the duration of different stages of projects with property buyouts, illustrating timing trade-offs. For each project involving at least one bought-out property, the time between the disaster event and project approval (x-axis) is compared to the time between project approval and project closeout (y-axis). Relatively few projects have short durations for both stages. Regression line: $y = -0.6x + 4.1$, $R^2=0.34$, $p \leq 0.001$. (**D**) The duration of buyout projects for which project closeout has occurred, by decade over program years 1989–2017. Density plots and means for total project duration are shown separately for 1989–1998 (940 projects), 1999–2008 (758 projects), and 2009–2017 (459 projects). *** indicates $p \leq 0.001$ for Kruskal–Wallis one-way analysis of variance across the damage timeframes, with pairwise comparisons of the most recent decade and prior decades statistically significant at the same level. Note that the fraction of HMGP buyout projects that are closed declines with each decade, from 98% for 1989–1998 to 64% for 2009–2017.
